# Thiamethoxam Resistance in *Aphis gossypii* Glover Relies on Multiple UDP-Glucuronosyltransferases

**DOI:** 10.3389/fphys.2018.00322

**Published:** 2018-04-03

**Authors:** Yiou Pan, Fayi Tian, Xiang Wei, Yongqiang Wu, Xiwu Gao, Jinghui Xi, Qingli Shang

**Affiliations:** ^1^College of Plant Science, Jilin University, Changchun, China; ^2^Department of Entomology, China Agricultural University, Beijing, China

**Keywords:** UDP-glucuronosyltransferase, insecticide resistance, *Aphis gossypii*, RNAi, thiamethoxam

## Abstract

Uridine diphosphate (UDP)-glycosyltransferases (UGTs) are major phase II enzymes that conjugate a variety of small lipophilic molecules with UDP sugars and alter them into more water-soluble metabolites. Therefore, glucosidation plays a major role in the inactivation and excretion of a great variety of both endogenous and exogenous compounds. In this study, two inhibitors of UGT enzymes, sulfinpyrazone and 5-nitrouracil, significantly increased the toxicity of thiamethoxam against the resistant strain of *Aphis gossypii*, which indicates that UGTs are involved in thiamethoxam resistance in the cotton aphid. Based on transcriptome data, 31 *A. gossypii UGTs* belonging to 11 families (UGT329, UGT330, UGT341, UGT342, UGT343, UGT344, UGT345, UGT348, UGT349, UGT350, and UGT351) were identified. Compared with the thiamethoxam-susceptible strain, the transcripts of 23 *UGTs* were elevated, and the transcripts of 13 *UGTs* (*UGT344J2, UGT348A2, UGT344D4, UGT341A4, UGT343B2, UGT342B2, UGT350C3, UGT344N2, UGT344A14, UGT344B4, UGT351A4, UGT344A11*, and *UGT349A2*) were increased by approximately 2.0-fold in the resistant cotton aphid. The suppression of selected *UGTs* significantly increased the insensitivity of resistant aphids to thiamethoxam, suggesting that the up-regulated *UGTs* might be associated with thiamethoxam tolerance. This study provides an overall view of the possible metabolic factor *UGTs* that are relevant to the development of insecticide resistance. The results might facilitate further work to validate the roles of these *UGTs* in thiamethoxam resistance.

## Introduction

Uridine diphosphate (UDP)-glycosyltransferases (UGTs, EC 2.4.1.17) catalyze the conjugation of a range of small lipophilic compounds with sugars to produce glycosides, which are soluble in water and can be efficiently excreted (Mackenzie et al., 1997). The protein structure of UGTs is divided into two main parts: the aglycone substrate-binding domain at the N-terminus and the UDP sugar donor-binding domain at the C-terminus (Magdalou et al., 2010). Glycoside conjugation is one of the most important metabolic pathways for the biotransformation of a number of lipophilic endogenous and exogenous compounds of xenobiotics and endobiotics (Bock, 2003, 2016; Bowles et al., 2005). Therefore, the glycosylation of toxins by UGTs is a particularly important detoxification mechanism (Heckel, 2014; Heidel-Fischer and Vogel, 2015). Long-term evolution has led to the development of sophisticated detoxification systems that allow organisms to resist various harmful substances occurring in the external environment. Insects use UDP-glucose as an activated sugar donor that is then transferred to UGTs, which are anchored in the endoplasmic reticulum (ER) (Ahn et al., 2012). Previous studies have indicated the involvement of insect UGTs in the detoxification of plant secondary xenobiotics in *Manduca sexta* (Ahmad and Hopkins, 1992), *Helicoverpa assulta* (Ahn et al., 2011a,b), *Spodoptera littoralis* (Wouters et al., 2014), *Helicoverpa armigera*, and *Heliothis virescens* (Krempl et al., 2016). In addition to plant secondary xenobiotic tolerance, insect UGTs might be involved in insecticide detoxification. Recent studies have demonstrated that the overexpression of *UGT2B17* (renamed *UGT33AA4*) is associated with chlorantraniliprole resistance in *Plutella xylostella* (Li et al., 2017a,b), and many studies have reported on cytochrome P450 monooxygenase-mediated insecticides resistance (Scott, 2008; Feyereisen, 2015). However, few studies have described the involvement of UGTs in the detoxification of insecticide resistance.

The cotton aphid, *Aphis gossypii* Glover (Hemiptera: Aphididae), is one of the most economically important insect pests in agriculture and has developed different levels of resistance to broad-spectrum insecticides, including organophosphates, pyrethroids, carbamates, and neonicotinoids (Denholm and Rowland, 1992; Shang et al., 2012; Chen et al., 2017). Thiamethoxam, a second-generation neonicotinoid insecticide that irreversibly binds to the nicotinic acetylcholine receptors (nAChR) of cells in the nervous system and interferes with the transmission of nerve impulses in insects (Casida and Durkin, 2013), and is effective for controlling resistant *A. gossypii* (Elbert et al., 2008). Research studies have indicated that enhanced detoxification caused by P450 gene overexpression accounts for neonicotinoids in *Bemisia tabaci, Myzus persicae*, and *Nilaparvata lugens* (Karunker et al., 2008, 2009; Puinean et al., 2010; Bao et al., 2016; Zhang et al., 2016). Consistent with these reports, our previous synergism analysis demonstrated that P450s are also involved in thiamethoxam resistance in *A*. *gossypii* (Wei et al., 2017). Whether *UGTs* are involved in insecticide resistance as well as P450-mediated resistance in *A. gossypii* has not been determined. The results of a synergism study illustrate that *UGTs* might be involved in the resistance present in thiamethoxam-resistant *A. gossypii*.

In this study, to clarify the potent roles of *UGTs* in thiamethoxam resistance in cotton aphids, (1) the *UGT* genes in the *A. gossypii* transcriptome were identified, and the phylogenetic relationships between these genes and their homologs in two other insects were analyzed; (2) the expression profiles of these *UGTs* in thiamethoxam-susceptible and thiamethoxam-resistant strains were analyzed by quantitative real-time polymerase chain reaction (qRT-PCR); and (3) the involvement of overexpressed *UGTs* in resistance was functionally tested by RNA interference (RNAi). Our data provide preliminary insights into the dynamic changes in the gene expression of *UGTs* and their involvement in thiamethoxam resistance. The results might facilitate further study of the functions in *UGTs* in the insecticide resistance of *A. gossypii*.

## Materials and methods

### Insects

Two cotton aphid (*A. gossypii*) strains were used in this study. One strain was resistant to thiamethoxam (ThR), and the other was susceptible to thiamethoxam (SS) (Pan et al., 2015). The SS strain was collected in 2008 from Jilin Province of China, where limited insecticides have been applied. The aphid species has been maintained without any insecticide treatment since its collection. The ThR strain was established from the SS population via consecutive selection with increased concentrations of thiamethoxam (LC_30_) via the leaf-dipping method. Both the resistant and susceptible strains were reared on cotton plants [*Gossypium hirsutum* (L.)] in the laboratory at 20–23°C with a photoperiod of 16:8 h (light:dark).

### Chemicals

Sulfinpyrazone (Sul) and 5-nitrouracil (5-Nul) were obtained from Sigma-Aldrich (St. Louis, MO, USA). Thiamethoxam (25% WDG) was purchased from Syngenta (Switzerland). The PrimeScript^TM^ First-Strand cDNA Synthesis kit, SYBR® Premix Ex Taq™ II (Tli RNaseH Plus), oligo(dT)_18_, Ex Taq DNA polymerase, RNase-free DNase I, RNase Inhibitor, DNA Marker DL2000, and agarose were purchased from TaKaRa (Dalian, China). The pGEM-T vector and the T7 RiboMAX™ Express RNAi System were purchased from Promega (USA). All the reagents were of the highest purity available.

### Bioassays

The synergistic effects of two UGT inhibitors, 5-nitrouracil (5-Nul) and sulfinpyrazone (Sul), on the toxicity of thiamethoxam to the SS and ThR strains were tested using a leaf dipping method, as described by Peng et al. (2016a) and Wei et al. (2017) with some modifications. The maximum sublethal doses of 5-Nul and Sul for the SS strain were determined using the bioassay method described by Wei et al. (2017). 5-Nul and Sul were used to prepare a series of concentrations (six or seven concentrations) with distilled water containing 0.05% (v/v) Triton X-100. The leaves were dipped for 15 s in the required concentration of insecticide or into 0.05% (v/v) Triton X-100 water (as the control treatment) and then placed in the shade and allowed to air dry. Bioassays were conducted by transferring at least 30 apterous adult aphids onto the treated cotton leaves obtained from each whole seedling. The bioassay samples were maintained in the laboratory at 20–23°C with a photoperiod of 16:8 h (light:dark). Three replicates were performed for each concentration, and the mortality was assessed after 3 days. The maximum dose that led to zero mortality in the SS strain was adopted as the maximum sublethal concentration in our study, and the maximum sublethal concentrations of 5-Nul and Sul were 400 mg/L. For synergism bioassays, apterous adult aphids were exposed to cotton leaves that were treated with the mixture of thiamethoxam with 5-Nul or Sul (final concentration of 400 mg/L). Three replicates were performed for each concentration, and the mortality was assessed after 3 days and used to estimate the synergistic effects of 5-Nul or Sul with thiamethoxam in both strains. The synergistic ratio was calculated using the conventional approach, which divides the LC_50_ without the synergist by the LC_50_ with the synergist. A probit analysis was conducted using POLO software (LeOra Software Inc., Berkeley, CA, USA).

### Nomenclature and phylogenetic analysis

The *UGT* sequences were derived from the transcriptome (the clean reads have been submitted to and are available from the National Center for Biotechnology Information (NCBI)/SRA database with SRA experiment accession number SRX683625) (Pan et al., 2015). These *UGT* sequences were named by the UGT Nomenclature Committee guidelines (Dr. Michael H. Court, Department of Veterinary Clinical Sciences, Washington State University, USA) using the following criteria: the gene symbol UGT, a family number, a subfamily letter, and an individual gene number. UGT families are defined at 40% amino acid sequence identity, and subfamilies are defined at 60% amino acid identity or greater (Mackenzie et al., 1997, 2005). Names were assigned to the *A. gossypii* sequences on this basis. The UGT predicted protein sequences from *H. armigera* and *Bombyx mori* were extracted from the UGT Nomenclature Committee (http://prime.vetmed.wsu.edu/resources/udp-glucuronsyltransferase-homepage)and analyzed with the *A. gossypii UGTs* via ClustalW alignment using MEGA 7 software (http://www.megasoftware.net/). The alignment results were used to build a consensus phylogenetic tree using the neighbor-joining method. Pairwise and multiple alignments were performed with a gap opening penalty of 10 and a gap extension penalty left of 0.2. A total of 1,000 bootstrap replications were performed, and branches with bootstrap values above 50% are indicated.

### Protein structure prediction

Multiple alignments of 10 overexpressed representative protein sequences from the *A. gossypii UGTs* were obtained via ClustalW, and the structural domains, such as the UGT signature motif, were detected by comparison with other sequences for which primary structures have been characterized. The signal peptides were predicted by SignalP 4.1 on the CBS Prediction Servers (http://www.cbs.dtu.dk/services/SignalP/). The C-terminal transmembrane domain was searched using TMHMM2.0 (http://www.cbs.dtu.dk/services/TMHMM).

### Total RNA isolation and cDNA synthesis

The total RNA from apterous adult aphids was extracted using TRIzol (Invitrogen, USA) according to the manufacturer's instructions and then treated with RNase-free DNase I (TaKaRa, Japan). The RNA samples were quantified by measuring the absorbance at 260 nm, and the quality was assessed via agarose gel electrophoresis. First-strand cDNA was synthesized from the total RNA using a PrimeScript^TM^ First-Strand cDNA Synthesis kit (TaKaRa, Japan) with oligo (dT)_18_ as a primer.

### Quantitative real-time PCR and data analysis

Quantitative real-time PCR was performed on an ABI 7500 system (Applied Biosystems) using SYBR® Premix Ex Taq™ II (Tli RNaseH Plus; TaKaRa, Japan) (Wei et al., 2017). Gene-specific primers for real-time PCR (Supplementary Table 1) were designed based on the *UGT* sequences (Supplementary Data 1) and synthesized by Sangon Biotech Co., Ltd. (Shanghai, China). The thermal cycling protocol included an initial denaturation at 95°C for 30 s followed by 40 cycles of 95°C for 5 s and 60°C for 34 s. The fluorescence signal was measured at the end of each extension step at 60°C. After the amplification, a dissociation step consisting of 95°C for 15 s, 60°C for 1 min and 95°C for 15 s was performed to confirm that only specific products were amplified. The glyceraldehyde-3-phosphate dehydrogenase (*GAPDH*) and elongation factor 1-alpha (*EF1a*) were used as internal reference genes for *A. gossypii* (Peng et al., 2016a,b). Relative gene expression was calculated using the 2^−ΔΔ*CT*^ method (Pfaffl, 2001). The experiment was independently performed three times for each strain. Significant differences were analyzed using GraphPad InStat3 statistical software (GraphPad Software, 2000, http://www.apponic.com/publisher/graphpad-software-18307/top-downloads/).

### Rearing on artificial diet and dsRNA feeding

The dsRNA design and synthesis methods were previously described by Peng et al. (2016a). Based on the *UGT* sequences (Supplementary Data 1) and the possible interference sites predicted with online prediction software (http://www.dkfz.de/signaling/e-rnai3/), we designed specific primers using DNAMAN 6.0 software (http://www.lynnon.com/dnaman.html). The gene fragments were amplified from cDNA and cloned into pGEM-T (Promega, USA). The purified plasmids served as templates for RNA synthesis using the T7 RiboMAX™ Express RNAi System (Promega, USA). *ECFP* dsRNA was used as the control and synthesized under the same conditions as the primers (Supplementary Table 1). The artificial diet and the rearing method used in this study were previously reported by Peng et al. (2016a,b). The diet was prepared in DEPC-treated water to ensure the absence of RNase activity. For the dsRNA feeding experiments, dsRNA was added to the artificial diet at a concentration of 100 ng/μL. An artificial diet containing dsRNA-*ECFP* was used as a control. Sixty adult apterous thiamethoxam-resistant *A. gossypii* were transferred onto the artificial diet for rearing. To analyze the efficiency of dsRNA knockdown on *UGT* expression, the aphids were fed an artificial diet containing dsRNA (100 ng/μL) for 48 h and then subjected to RT-qPCR. To assess the sensitivity of the cotton aphids to thiamethoxam after RNAi of *UGT*, 80 resistant adult aphids were transferred to the artificial diet containing thiamethoxam (1.0 mg/L) mixed with dsRNA*-UGT* (100 ng/μL), and dsRNA*-ECFP* was used as the control. The mortality of the cotton aphids was recorded after 48 h. Each treatment included three replicates (80 aphids were used in each replication).

## Results

### Thiamethoxam toxicity and synergism bioassays

The probit analyses of thiamethoxam toxicity and synergism bioassays of *A. gossypii* are summarized in Table 1. 5-Nul and Sul increased the thiamethoxam toxicity in the ThR strain by 9.38- and 10.31-fold, respectively. These results indicate that UDP-glucuronosyltransferases are involved in thiamethoxam resistance in the ThR strain at the observed resistance state.

**Table 1:** Synergistic effects of Sul and 5-Nul on the toxicity of thiamethoxam in the SS and ThR strains.

**Strain**	**Thiamethoxam/Thiamethoxam + synergist**	**Fit of probit line^a^**			**LC_50_ (95% CL^b^) (mg L^−1^)**	**SR^c^ (at 95% CL^b^)**
		**Slope ±SE**	**χ^2^**	***df***		
SS	Thiamethoxam^*^	2.33 ± 0.11	16.90	16	2.85 (1.74–4.24)^*^	–
	Thiamethoxam+Sul	3.45 ± 0.33	9.75	16	2.41 (2.119–2.72)	1.18
	Thiamethoxam+5-Nul	3.46 ± 0.27	23.94	16	2.99 (2.59–3.41)	0.95
ThR	Thiamethoxam^*^	1.56 ± 0.11	31.76	16	55.16 (30.27–161.05)^*^	–
	Thiamethoxam+Sul	2.04 ± 0.21	14.36	16	5.88 (4.93–6.84)	9.38
	Thiamethoxam+5-Nul	2.94 ± 0.21	26.26	16	5.35 (4.54–6.23)	10.31

* Data obtained from Pan et al. (2015).

a Probit model fitted using POLO-PC (LeOra Software, 1987).

b Confidence limits.

c *SR (synergism ratio) = LC_50_ of thiamethoxam/LC_50_ of thiamethoxam with synergist*.

### Identification and phylogenetic analysis of the *A. gossypii* UGTs

**Figure 1:**
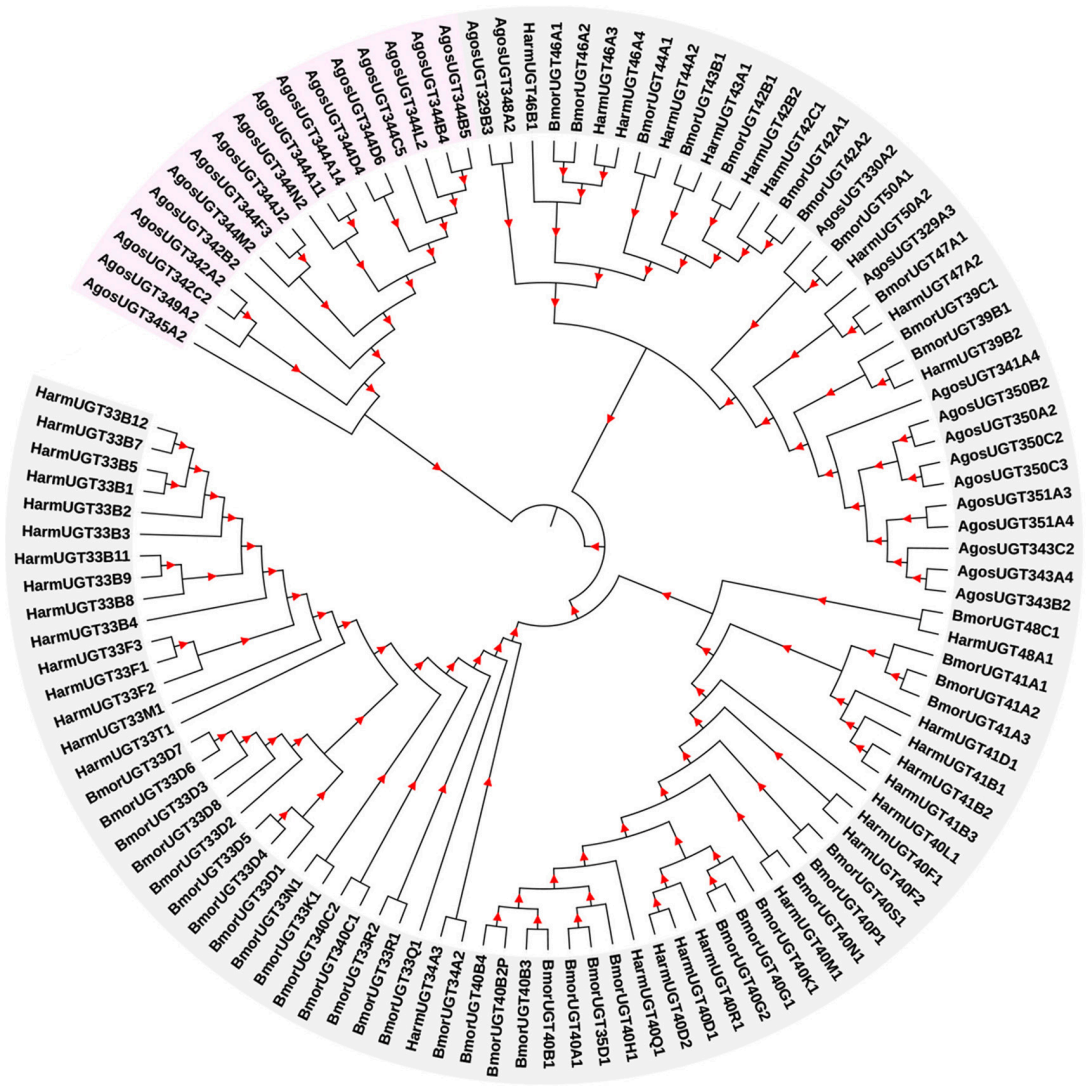
Phylogenetic analysis of *A. gossypii* UGT genes with other orthologs. The phylogram was generated using the maximum likelihood method in MEGA 7, and bootstrap values were calculated based on 1,000 replicates with a cutoff of <50%. The sequences used for constructing the tree and their corresponding GenBank accession numbers are listed in Supplementary Data 1.

Based on the transcriptome data from *A. gossypii*, the full lengths of 31 *UGT* genes were identified, and these *UGT* genes were named by the UGT Nomenclature Committee (Dr. Michael H. Court, Department of Veterinary Clinical Sciences, Washington State University, USA). The GenBank accession numbers are listed in Supplementary Data 1. To construct a phylogenetic tree (Figure 1), a total of 83 *UGT* gene sequences of *H. armigera* and *B*. *mori* from the *UGT* Nomenclature Committee (Supplementary Data 1) and 31 *UGT* gene sequences from our cotton aphid transcriptome were used in the phylogenetic tree. ClustalW alignments performed using MEGA 7 software (http://www.megasoftware.net/) were used to align the amino acid sequences, and the neighbor-joining method with 1,000 bootstrap replicates was used to construct the phylogenetic trees.

The 31 *A. gossypii UGTs* were distributed into 11 families, namely, the UGT344 (12 *UGTs*), UGT350 (4), UGT342 (3), UGT343 (3), UGT329 (2), UGT351 (2), UGT330 (1), UGT341 (1), UGT345 (1), UGT348 (1), and UGT349 (1) families. The number of *UGT* genes in *A. gossypii* (31 *UGTs*) was less than that in *H. armigera* and *B. mori*, and this finding is related to the lack of a complete genome sequence for *A. gossypii*.

### Structural motifs of the *A. gossypii* UGT proteins

**Figure 2:**
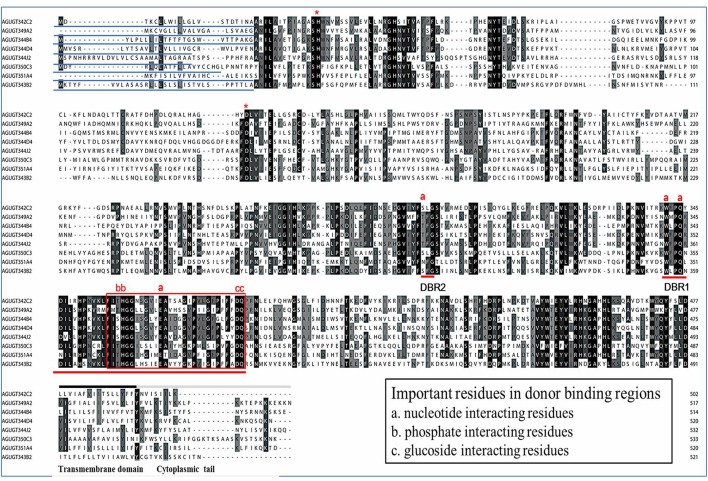
Alignment of the amino acid sequences of eight UGT genes from *A. gossypii*. The predicted signal peptides are underlined, and the UGT signature motif is boxed. The transmembrane domains and cytoplasmic tails in the C-terminus are underlined by black and gray lines above the alignment, respectively. The conserved catalytic residues, H and D, are indicated by * above the alignment. DBR refers to the donor-binding region, and several important residues interacting with the sugar donor are indicated (a, b, or c) above the alignment.

Multiple alignments of eight representative *A. gossypii* UGT amino acid sequences revealed two major domains: the highly variable N-terminal substrate-binding domain and the conserved C-terminal sugar donor-binding domain (Figure 2) (Ahn et al., 2012). All *A. gossypii* UGTs consisted of a different length amino acid signal peptide found at the N-terminal end, which is presumably cleaved after integration into the ER compartment. The two predicted sugar donor-binding regions (DBR1 and DBR2), important residues interacting with the sugar donor, and catalytic residues were conserved. The UGT motif signature sequences [also called the plant secondary product glycosyltransferase (PSPG) motif in plants], (FVA)-(LIVMF)-(TS)-(HQ)-(SGAC)-G-X(2)-(STG)-X(2)-(DE)-X(6)-P-(LIVMFA)-(LIVMFA)-X(2)-P-(LMVFIQ)-X(2)-(DE)-Q (where X is any amino acid), was found in the middle of the C-terminal domain, which shows higher conservation (Mackenzie et al., 1997; Ahn et al., 2012). The alignment data suggested that most *A. gossypii* UGTs were active proteins.

### Expression profiling of *A. gossypii UGT* genes in the resistant and susceptible strains

**Figure 3:**
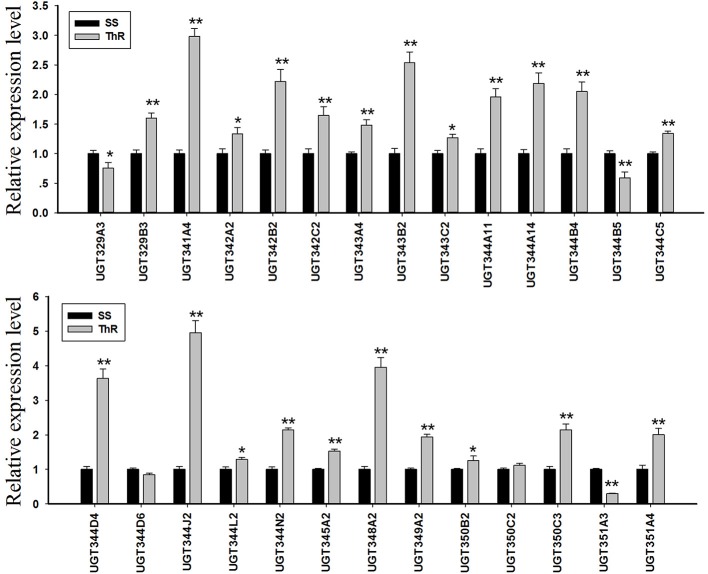
Transcription levels of *UGTs* in the SS and SR strains determined by real-time PCR. GAPDH and EF1a were used as internal reference genes. The error bars indicate 95% confidence intervals (*n* = 3). *P < 0.05 difference, as determined by Student's *t-test*. **P < 0.01 difference, as determined by Student's *t-test*.

The quantitative real-time PCR results indicated that the transcripts of 23 *UGT* genes of the 27 determinate genes were elevated, and the transcripts of 13 *UGT* genes were increased by approximately 2.0-fold or greater in the thiamethoxam-resistant cotton aphid compared with the susceptible aphids. Specifically, the mRNA levels of *UGT344J2, UGT348A2, UGT344D4, UGT341A4, UGT343B2, UGT342B2, UGT350C3, UGT344N2, UGT344A14, UGT344B4, UGT351A4, UGT344A11*, and *UGT349A2* were increased to 4.96, 3.95, 3.64, 2.98, 2.54, 2.22, 2.14, 2.14, 2.18, 2.05, 2.00, 1.96, and 1.94-fold, respectively. In contrast, the transcripts of *UGT344D6, UGT329A3, UGT344B5*, and *UGT351A3* were down-regulated in the ThR strain compared with the SS strain, and the *UGT351A3* level was decreased by 0.29-fold (Figure 3).

### Suppression of *UGT* transcripts increases thiamethoxam toxicity

**Figure 4:**
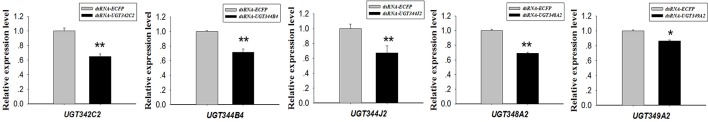
dsRNA-mediated suppression of *UGT* transcripts in *A. gossypii*. dsRNA-mediated suppression of *UGT342C2, UGT344B4, UGT344J2, UGT348A2*, and *UGT349A2* transcripts in resistant aphids fed an artificial diet with the corresponding dsRNA (100 ng/μL). The error bars indicate 95% confidence intervals (*n* = 3). *P < 0.05 difference, as determined by Student's *t-test*. **P < 0.01 difference, as determined by Student's *t-test*.

**Figure 5:**
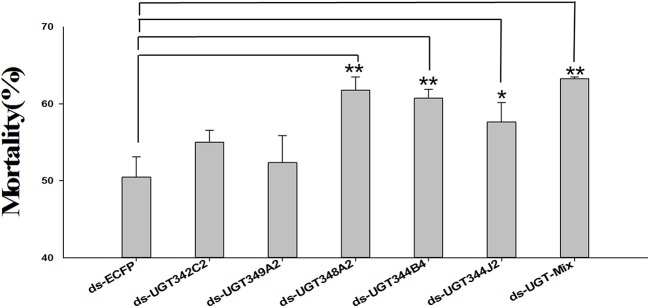
Effects of the knockdown *UGT* on thiamethoxam toxicity in *A. gossypii*. Mean mortality ± SE (*n* = 3) of resistant cotton aphids that were fed thiamethoxam (1.0 mg/L) and a dsRNA-*UGT* (100 ng/μL) for 48 h. Each treatment included three replicates, and 80 adults of the resistant aphid strain were used in each replication. The error bars indicate 95% confidence intervals (*n* = 3). *P < 0.05 difference, as determined by Student's *t-test*. **P < 0.01 difference, as determined by Student's *t-test*.

An orally delivered dsRNA method for RNAi was performed to elucidate the relationship between the overexpression of massive *UGT* genes and thiamethoxam resistance. Because many *UGT* genes were up-regulated in the ThR strain, we chose five *UGT* genes for the RNAi experiment. Under the RNAi treatments, the expression levels of *UGT342C2, UGT344B4, UGT344J2, UGT348A2*, and *UGT349A2* were reduced to 0.65-, 0.72-, 0.67-, 0.65-, 0.69-, and 0.86-fold in the corresponding dsRNA*-UGT-*treated (100 ng/μL) aphids after 48 h of treatment compared with the control expression levels (Figure 4). The mortality increased from 50.46% in the control to 55.03, 60.74, 57.66, 61.79, 52.38, and 63.27% in the aphids fed dsRNA*-UGT342C2*, dsRNA*-UGT344B4*, dsRNA*-UGT344J2*, dsRNA*-UGT348A2*, dsRNA*-UGT349A2*, and dsRNA-Mix (the ratio of six dsRNA-*UGT* is 1:1:1:1:1:1:1) under the 1.0 mg/L thiamethoxam treatments, respectively (Figure 5).

## Discussion

Due to the extensive use of the neonicotinoid insecticide imidacloprid for controlling cotton aphids in the field, the resistance to imidacloprid ranged from 1.48 to >1,200-fold among different *A*. *gossypii* populations collected from various Bt cotton planting areas in China in 2014, and the LC_50_-value of imidacloprid was >5,000 mg/L in the population from Yuncheng of Shanxi Province (Chen et al., 2017). Thiamethoxam has been used as an alternative neonicotinoid insecticide for the control of cotton aphids. Our previous synergism assay showed that phase I enzyme P450s (acting directly on the toxin molecule) are involved in thiamethoxam resistance (Wei et al., 2017). In addition to P450-mediated detoxification resistance, the roles of the phase II enzyme UGTs (which conjugate endogenous molecules to the toxins) in thiamethoxam resistance remains unknown. To clarify the roles of UGTs in thiamethoxam tolerance, the UGT inhibitors 5-nitrouracil and sulfinpyrazone were used in a synergism assay, and the results illustrated that these two UGT inhibitors significantly increased thiamethoxam toxicity in the ThR strain (Table 1), suggesting that UGTs, in addition to P450, are involved in thiamethoxam resistance. Therefore, glycosylation by UGTs might play an important role in the detoxification of xenobiotics in the cotton aphid. Notably, information regarding UGTs in the cotton aphid has not been available until now.

In this study, 31 *UGT* genes were identified from the transcriptome data of *A. gossypii* (Pan et al., 2015). The *A. gossypii UGTs* were distributed into 11 families: UGT329, UGT330, UGT341, UGT342, UGT343, UGT344, UGT345, UGT348, UGT349, UGT350, and UGT351 (Figure 1). The fewer number of *UGT* genes identified in *A. gossypii* (31 *UGTs*) compared with those found in *H. armigera* and *B. mori* might be due to the lack of a complete genome sequence for *A. gossypii*. The UGT protein structure is divided into two main parts: the aglycone substrate-binding domain in the N-terminus and the UDP sugar donor-binding domain in the C-terminus (Meech and Mackenzie, 1997; Meech et al., 2012). Alignments of the *A. gossypii* UGT amino acid sequences showed conserved domains, including the sugar donor-binding region (DBR1 and DBR2), important residues interacting with the sugar donor and catalytic residues, and the UGT motif signature sequences [(FVA)-(LIVMF)-(TS)-(HQ)-(SGAC)-G-X(2)-(STG)-X(2)-(DE)-X(6)-P-(LIVMFA)-(LIVMFA)-X(2)-P-(LMVFIQ)-X(2)-(DE)-Q] (Figure 2) (Mackenzie et al., 1997; Ahn et al., 2012). These findings suggested that *A. gossypii* UGTs were likely active proteins, which is similar to observations in mammals. Insect UGTs are capable of detoxifying plant secondary compounds. For example, the stereoselective reglucosylation of benzoxazinoid by UGT represents a detoxification strategy in *S*. *littoralis* (Wouters et al., 2014). UGTs are capable of glycosylating gossypol primarily to the diglycosylated gossypol isomer 5, which is a crucial step in gossypol detoxification in *H. armigera* (Krempl et al., 2016). Insect UGTs mediate plant xenobiotic tolerance and have also been reported to be involved in insecticide resistance. The ingestion of dsRNA, which successfully silences overexpressed *UGTs*, significantly increases the susceptibility of resistant *Leptinotarsa decemlineata* to imidacloprid (Kaplanoglu et al., 2017). In *P*. *xylostella*, the overexpression of *UGTs* is associated with chlorantraniliprole resistance (Li et al., 2017a,b). In this study, a screening of the expression profile of *UGTs* revealed that the expression of 13 *UGT* genes was increased by nearly 2-fold or more in the ThR strain compared with the SS strain (Figure 3), suggesting that multiple up-regulated *UGTs* might be associated with thiamethoxam resistance. To verify the influence of *UGT* gene overexpression on the susceptibility to thiamethoxam in *A. gossypii*, we performed RNAi via a dsRNA oral feeding method (Peng et al., 2016a,b) and found that the RNAi of the overexpressed *UGT* genes could have resulted in increased thiamethoxam susceptibility in the resistant cotton aphids (Figure 5). This result further confirmed that enzymes encoded by these overexpressed *UGTs* might contribute to the detoxification of thiamethoxam by glycosylation in *A. gossypii*. In conclusion, this study provides insights into the potential roles of *UGTs* in thiamethoxam resistance. These results should be useful for understanding thiamethoxam resistance mechanisms.

## Author contributions

All the authors listed have made a substantial, direct and intellectual contribution to the work and have approved its publication. YP and FT: designed and performed most of the experiments. XW and YW: performed the RNAi assays. XG and JX: revised the manuscript. QS: designed the experiments and wrote the manuscript.

### Conflict of interest statement

The authors declare that the research was conducted in the absence of any commercial or financial relationships that could be construed as a potential conflict of interest.

## Supplementary material

Supplementary Table 1 Primers used in the experiments. dsRNA, double-stranded RNA; EF1a, elongation factor 1-alpha; F, forward; GAPDH, glyceraldehyde-3-phosphate dehydrogenase; ORF, open reading frame; R, reverse. The lowercase letters indicate the T7 RNA polymerase promoter.

Supplementary Data 1 Gene sequences of *A. gossypii UGTs* and other insect orthologs.
