# Supplementary material for: Thiamethoxam Resistance in Aphis gossypii Glover Relies on Multiple UDP-Glucuronosyltransferases
Source: Front Physiol. 2018 Apr 3;9:322. doi: 10.3389/fphys.2018.00322 (PMC5893893; doi:10.3389/fphys.2018.00322)
Supplement: Supplementary Table 1 — Primers used in the experiments. dsRNA, double-stranded RNA; EF1a, elongation factor 1-alpha; F, forward; GAPDH, glyceraldehyde-3-phosphate dehydrogenase; ORF, open reading frame; R, reverse. The lowercase letters indicate the T7 RNA polymerase promoter. [file Table1.DOC]

**Table 1. Primers used in this study.**

| **Primer name** | **Sequence (5'-3')** | **Application** |
| --- | --- | --- |
| GAPDH-F  GAPDH-R  EF1a-F  EF1a-R  UGT344N2-F  UGT344N2-R  UGT344C5-F  UGT344C5-R  UGT342C2-F  UGT342C2-R  UGT350B2-F  UGT350B2-R  UGT344L2-F  UGT344L2-R  UGT350C2-F  UGT350C2-R  UGT343C2-F  UGT343C2-R  UGT342A2-F  UGT342A2-R  UGT329B3-F  UGT329B3-R  UGT344B4-F  UGT344B4-R  UGT350C3-F  UGT350C3-R  UGT345A2-F  UGT345A2-R  UGT351A3-F  UGT351A3-R  UGT348A2-F  UGT348A2-R  UGT343A4-F  UGT343A4-R  UGT329A3-F  UGT329A3-R  UGT344A11-F  UGT344A11-R  UGT351A4-F  UGT351A4-R  UGT344D4-F  UGT344D4-R  UGT344D6-F  UGT344D6-R  UGT349A2-F  UGT349A2-R  UGT344A14-F  UGT344A14-R  UGT342B2-F  UGT342B2-R  UGT343B2-F  UGT343B2-R  UGT344B5-F  UGT344B5-R  UGT344F3-F  UGT344F3-R  UGT344J2-F  UGT344J2-R  UGT341A4 qPCR-F  UGT341A4 qPCR-R  UGT342C2-dsF1  UGT342C2-dsR1  UGT342C2-dsF2  UGT342C2-dsR2  UGT344B4 -dsF1  UGT344B4 -dsR1  UGT344B4 –dsF2  UGT344B4 –dsR2  UGT348A2-dsF1  UGT348A2-dsR1  UGT348A2-dsF2  UGT348A2-dsR2  UGT349A2-dsF1  UGT349A2-dsR1  UGT349A2-dsF2  UGT349A2-dsR2  UGT344J2-dsF1  UGT344J2-dsR1  UGT344J2-dsF2  UGT344J2-dsR2  ECFP-dsRNA-F1  ECFP- dsRNA-R1  ECFP- dsRNA-F2  ECFP- dsRNA-R2 | AACAGTTTTTTGAGTGGCGGT  TGGTGTCAACTTGGATGCGTA  CAGTCCCAGTAGGTCGTGTTG TCAGTGGTGATGTTAGCAGGT  AAGGAGCCATTGGAATGTTA  CTGGAATCGGTCATTATACTCTT  CCTCAGCGTGATATACTTCTGCATC  CATTCCCATTCCTGCTT  AAACGACGCTCAACTAACCA  GGAGCCGAGCAATTCTGT  CATCTATTCCAAATGCTGGTG  TGACGGTCGTGTCTCCC  TCCGCCGTTCCCAAGAC  CCACCGACACTAACAACATTCG  AAAATGCCCAAGGAAACAG  GGGAACTCCGTGATAGACG  ATCCGTCCACTTTACCA  TGAATCCCACTTCCACA  CAAAGCCACTGTTGCCTAAT  AATACGCTGGTGCTGTTTC  CACATCGTGAGTAACGGAGAA  CCATGCAATAAACGGAGC  GGTTCGTGGGTCACTACTCC  TTGCCCATCTAGTATCTTCTCA  GTGTCGCAGTGGCAAGG  CGTTCTGGAGCATCGTCT  GCGGCATACACCTGAACC  CATAGCCACCAAAGAACCAA  TTATTTCGTGAACGGAGTATGT  TGTTTGCCCACCGAGATA  ACCACCGCTCAGAGTCAT  TTGGCGTTTCCCGTTAT  TCATAACTCACGGAGGATTG  GCACTTCTTTGACGGCATT  CGGGTACAGCCATCACA  ACCGAATAGAGCCGTCAA  GCCAAGCACGGAAGTCA  ACGCACTCGGACACCAG  TGGAATTGGCACATAGAGGT  TTCTGGTTATTGCCTGAGATAGT  TACTCGGATTTCCTTTGTTCT  TCAGGTCCAAGGCAATAGC  GTCAGCCCATCTATTATCTTCC  GGCGGGTTTCAGGTGTAT  CGGTGGACTGTTAGGGGTA  CGCATTTATAGCGTAACTGTCA  GGGACTTGAAGGTTAGGG  ATCGGTGACGGAATGAC  CGTTACAAGAGGCATTTGCT  GATGATGGCTTCATTTATACCAG  CCGTCAATGGTCTGGGTC  TGAGCGTTCATCAGCGTTA  ATTTCAATAGTGATTTGGGTTCT  CATAGCTCCTCCTAAAGTTGTG  TCGTGTCGTCCGAGTGC  CGTACCGAGCGAACAGC  TGGGCTTTCCGCTTTACT  GACTCTTGAGGCGTCATCG  CATTCTTCCCGTTGCCA  ACGGACTGACTACGGTTACG  GGTGtaatacgactcactataggGATTCGTTCAACAGTCCGTC  TGGTAAATCTTCCATCCTGAC  GATTCGTTCAACAGTCCGTC  GGTGtaatacgactcactataggTGGTAAATCTTCCATCCTGAC  GGTGtaatacgactcactataggTATCGGATTGCGTGTCGTA  GGATTTGTGCGAGAGATTC  TATCGGATTGCGTGTCGTA  GGTGtaatacgactcactataggGGATTTGTGCGAGAGATTC  GGTGtaatacgactcactataggCGGTCTGGCGAAGATGATG  GCAGCGAGGGATAGGGTAAA  CGGTCTGGCGAAGATGATG  GGTGtaatacgactcactataggGCAGCGAGGGATAGGGTAAA  GGTGtaatacgactcactataggCGTTGCTCCACAGCGACAT  TCCACAAGACTCTGATAGGTAA  CGTTGCTCCACAGCGACAT  GGTGtaatacgactcactataggTCCACAAGACTCTGATAGGTAA  GGTGtaatacgactcactataggGTGGCGTTCACCTCTTACCC  GGTGGCAGTAACATCGTAGGCT  GTGGCGTTCACCTCTTACCC  GGTGtaatacgactcactataggGGTGGCAGTAACATCGTAGGCT  GGTG taatacgactcactataggTTACGCCAAGCTTGCATGCCT  ACTCCAGCAGGACCATGTGATC  TTACGCCAAG CTTGCATGCCT  GGTG taatacgactcactataggACTCCAGCAGGACCATGTGATC | Real-Time PCR  Real-Time PCR  Real-Time PCR  Real-Time PCR  Real-Time PCR  Real-Time PCR  Real-Time PCR  Real-Time PCR  Real-Time PCR  Real-Time PCR  Real-Time PCR  Real-Time PCR  Real-Time PCR  Real-Time PCR  Real-Time PCR  Real-Time PCR  Real-Time PCR  Real-Time PCR  Real-Time PCR  Real-Time PCR  Real-Time PCR  Real-Time PCR  Real-Time PCR  Real-Time PCR  Real-Time PCR  Real-Time PCR  Real-Time PCR  Real-Time PCR  Real-Time PCR  Real-Time PCR  Real-Time PCR  Real-Time PCR  Real-Time PCR  Real-Time PCR  Real-Time PCR  Real-Time PCR  Real-Time PCR  Real-Time PCR  Real-Time PCR  Real-Time PCR  Real-Time PCR  Real-Time PCR  Real-Time PCR  Real-Time PCR  Real-Time PCR  Real-Time PCR  Real-Time PCR  Real-Time PCR  Real-Time PCR  Real-Time PCR  Real-Time PCR  Real-Time PCR  Real-Time PCR  Real-Time PCR  Real-Time PCR  Real-Time PCR  Real-Time PCR  Real-Time PCR  Real-Time PCR  Real-Time PCR  dsRNA synthesis  dsRNA synthesis  dsRNA synthesis  dsRNA synthesis  dsRNA synthesis  dsRNA synthesis  dsRNA synthesis  dsRNA synthesis  dsRNA synthesis  dsRNA synthesis  dsRNA synthesis  dsRNA synthesis  dsRNA synthesis  dsRNA synthesis  dsRNA synthesis  dsRNA synthesis  dsRNA synthesis  dsRNA synthesis  dsRNA synthesis  dsRNA synthesis  dsRNA synthesis  dsRNA synthesis  dsRNA synthesis  dsRNA synthesis |

dsRNA, double-stranded RNA; EF1a, elongation factor 1-alpha; F, forward; GAPDH, glyceraldehyde-3-phosphate dehydrogenase; ORF, open reading frame; R, reverse. The lowercase letters indicate the T7 RNA polymerase promoter.
